# Byakangelicin protects against carbon tetrachloride–induced liver injury and fibrosis in mice

**DOI:** 10.1111/jcmm.15493

**Published:** 2020-07-09

**Authors:** Xiaohe Li, Shuaibo Shao, Hailong Li, Zhun Bi, Shanshan Zhang, Yiying Wei, Jiakun Bai, Ruotong Zhang, Xiaoyang Ma, Bowei Ma, Liang Zhang, Chunfeng Xie, Wen Ning, Honggang Zhou, Cheng Yang

**Affiliations:** ^1^ State Key Laboratory of Medicinal Chemical Biology College of Pharmacy and Tianjin Key Laboratory of Molecular Drug Research Nankai University Tianjin China; ^2^ Tianjin Key Laboratory of Molecular Drug Research Tianjin International Joint Academy of Biomedicine Tianjin China; ^3^ Department of Thoracic Surgery Tian Jin First Central Hospital Tianjin China; ^4^ College of Life Sciences Nankai University Tianjin China

**Keywords:** apoptosis, byakangelicin, hepatic stellate cells, liver fibrosis, liver injury, PDGF, TGF‐β

## Abstract

Liver fibrosis is a disease caused by long‐term damage that is related to a number of factors. The current research on the treatment of liver fibrosis mainly focuses on the activation of hepatic stellate cell, in addition to protecting liver cells. byakangelicin has certain anti‐inflammatory ability, but its effect on liver fibrosis is unclear. This study aims to explore whether byakangelicin plays a role in the development of liver fibrosis and to explore its mechanism. We determined that byakangelicin has a certain ability to resist fibrosis and reduce liver cell damage in a model of carbon tetrachloride–induced liver fibrosis in mice. Thereafter, we performed further verification in vitro. The signalling pathways of two important pro‐fibrotic cytokines, transforming growth factor‐β and platelet‐derived growth factor, were studied. Results showed that byakangelicin can inhibit related pathways. According to the hepatoprotective effect of byakangelicin observed in animal experiments, we studied the effect of byakangelicin on 4‐HNE–induced hepatocyte (HepG2) apoptosis and explored its related pathways. The results showed that byakangelicin could attenuate 4‐HNE–induced hepatocyte apoptosis via inhibiting ASK‐1/JNK signalling. In conclusion, byakangelicin could improve carbon tetrachloride–induced liver fibrosis and liver injury by inhibiting hepatic stellate cell proliferation and activation and suppressing hepatocyte apoptosis.

## 1 INTRODUCTION

Liver fibrosis is a pathological process resulting from excessive accumulation of extracellular matrix protein and difficult degradation, which occurs because of continuous liver damage and abnormal repair programme.^1^ The common causes of liver fibrosis are hepatocyte injury induced by many factors, such as ethanol, parasites, HBV and HCV infection, non‐alcoholic fatty liver disease, drug treatment and inborn errors of metabolism.^2^ With the development of liver fibrosis, the extracellular matrix protein deposition becomes serious, and liver fibrosis develops into cirrhosis, which even increases the risk of liver cancer.^3^ Liver organ transplantation is an effective treatment for liver fibrosis; however, the transplantation as a treatment is subjected to conditions.^4^ Therefore, there is an urgent need for anti‐fibrotic drugs.

There are two important events in liver fibrosis that involve two types of cells. First, the most crucial event in the progression of liver fibrosis is the activation of hepatic stellate cell.^5^ Hepatic stellate cells, first described by Kupffer in 1876, are perisinusoidal cells that store lipid droplets.^6^ Hepatic stellate cell accounts for 15% of the resident cell population located in the sub‐endothelial space of Disse, between the basolateral surface of hepatocytes and the anti‐luminal side of sinusoidal endothelial cells.^7^ In normal liver fibrosis, hepatic stellate cells are at the quiescent state, whereas after liver injury, hepatic stellate cells lose lipid droplets and begin to activate, eventually differentiating into myofibroblasts. The activated hepatic stellate cell characterized by enhanced extracellular matrix is proliferative and chemotactic and secrete pro‐fibrotic protein or cytokine.^8^ Transforming growth factor (TGF)‐β is one of the most important cytokines in liver fibrosis.^9^, ^10^, ^11^ The classical downstream signalling pathway of TGF‐β is the Smad pathway. Apart from this, platelet‐derived growth factor (PDGF) is also a potent crude mitogen in hepatic stellate cell. PDGF can transfer signals to cells in a variety of ways, including Ras/ERK, PI3K/AKT and JAK/STAT.^12^, ^13^, ^14^

The apoptosis of hepatocytes is another important event in the process of liver fibrosis because it triggers liver fibrosis and further promotes the deterioration of fibrosis. Takehara reported that knocking out anti‐apoptotic BCL‐XL leads to hepatocyte apoptosis, which also causes liver fibrosis.^15^ By contrast, inhibiting hepatocyte apoptosis by interfering Fas with siRNA could reduce liver fibrosis.^16^ In addition, hepatocyte apoptotic body can be phagocytized by hepatic stellate cell and directly stimulate hepatic stellate activation to cause liver fibrosis.^17^, ^18^

Moreover, in the initial stage of liver fibrosis, the occurrence of inflammatory response is one of the important events, and the large amount of reactive oxygen species (ROS), producing oxidative stress, is an important feature. The incentives for oxidative stress prompted Keap‐1/Nrf‐2's antioxidant defence system to up‐regulate cell protective genes, which is considered to have a potential target for reducing chronic liver injury in liver fibrosis.^19^ Besides, nuclear factor‐κB (NF‐κΒ) is another redox‐sensitive signal transduction pathway, which plays a key role in the development of liver fibrosis by mediating inflammation.^20^

Byakangelicin is one of the furanocoumarins extracted from the root of byakangelicin and has protective effect on hepatocyte HepG2.^21^ In China and Korea, byakangelicin is used as traditional medicine for treating toothache, headache and so on.^22^ In addition, studies have shown that byakangelicin has certain anti‐inflammatory activity, which could inhibit 48/80‐induced histamine elevation and reduce cyclooxygenase 2 and inhibit PGE2.^23^, ^24^ Based on the important role of the above events in liver fibrosis, we first investigated the anti‐fibrotic mechanism of byakangelicin in liver fibrosis from pro‐fibrosis pathway and anti‐apoptosis pathway.

## 2 METHODS

### 2.1 Reagents and antibodies

Byakangelicin (purity ≥ 98.5%) was obtained from Push Bio‐technology Co., Ltd. Silibinin (purity > 97%) was obtained from Meilunbio Co., Ltd. Olive oil and carbon tetrachloride were obtained from Shanghai Macklin Biochemical Co., Ltd. byakangelicin was dissolved in dimethylsulphoxide (DMSO) from Sigma‐Aldrich Corporation for cell experiment. Recombinant human PDGF‐BB was obtained from R&D system. Recombinant Human TGF‐β1 was obtained from PeproTtech. Primary antibodies including anti‐α‐smooth muscle actin (α‐SMA), anti‐collagen Ⅰ (COL‐1), anti‐GAPDH and anti‐p‐Stat3, Stat3, β‐tubulin, cyclin D1, P53, p‐ASK‐1, ASK‐1, IL‐1β, NF‐κB and Nrf‐2 were acquired from Affinity Biosciences; anti‐p‐Smad3, Smad3, p‐PDGFR, PDGFR, p‐ERK, ERK, p‐AKT, AKT, PARP, cleaved caspase‐3 and caspase‐3 were received from (Cell Signaling Technology); anti‐p‐JNK, JNK from Signalway Antibody LLC; and anti‐4‐HNE from Abcam. Reagents and antibodies are prepared according to the instructions.

### 2.2 Animal model

Six‐week‐old male C57BL/6 mice (18‐22 g) were obtained from the Laboratory Animal Center, Academy of Military Medical Sciences of People's Liberation Army (Beijing, China). In the liver fibrosis model group and medication administration group, carbon tetrachloride (1 mL/kg) dissolved in olive oil was administered three times a week via intraperitoneal injection for eight weeks. The control group used the same dose and frequency of the model group. Silibinin, also known as silybin, serves as the positive control,^25^, ^26^ and a number of studies have confirmed its anti‐fibrosis effect.^27^, ^28^, ^29^ In the medication administration group, byakangelicin (100 mg/kg) and silibinin (100 mg/kg) were administered after four weeks through oral gavage until the end of eight weeks. After eight weeks, mice were anaesthetized by using chloral hydrate before being killed. Blood from mice was collected for alanine aminotransferase (ALT) and aspartate aminotransferase (AST) assay. The left lobe of the liver was placed in 10% formalin and then embedded with paraffin. Medium lobes of the liver were collected to dry for the assay of hydroxyproline content. The remaining lobe of the liver was rapidly placed in liquid nitrogen for protein and RNA extraction.

### 2.3 Histological analysis of the liver

The left lobe of mouse liver was placed in 10% formaldehyde, dehydrated and then embedded in paraffin blocks. It was sectioned to 4 µm thickness for staining using Masson's Trichrome Stain Kit and Picrosirius Red from Solarbio Technology Co., Ltd. Moreover, we also used the paraffin section for dewaxing to water and antigen repair. After the liver slices were incubated with primary antibody, we used UltraSensitive™ SP (Mouse/Rabbit) IHC Kit from Maixin Biotech. Co., Ltd for staining. We used the TUNEL staining method by using the Colorimetric TUNEL Apoptosis Assay Kit (Beyotime Biotechnology) to observe the apoptosis of liver tissue. We randomly selected the field of view to take pictures using upright transmission fluorescence 162 microscope (Olympus) and analysed the photos using Image‐Pro Plus Version 6.0 (Media 163 Cybernetics, Inc American). Apart from these, we used the alanine aminotransferase assay kit and aspartate aminotransferase assay kit from Nanjing Jiancheng Bioengineering Institute (Nanjing, China) for ALT and AST content determination, respectively.

### 2.4 Hydroxyproline content determination

After separating the middle lobe of the liver, we placed it in an ampoule for drying and added hydrochloric acid. Upon completion of the steps above, we adjusted the pH of mixture to 6.5‐8.0 and filtered it. Finally, we set the volume to 10 mL with phosphate‐buffered saline (PBS) by adding 350 µL H_2_O, 50 µL sample and 200 µL chloramine‐T into each tube in sequence. We vortexed the tube and incubated it at room temperature for 20 minutes. Then, we added 200 µL perchloric acid to the tube with vortexing and incubated the solution for 5 minutes at room temperature. After adding 200 µL P‐DMAB and incubation at 60°C in a water bath for 20 minutes, we transferred 200 µL of the sample to 96‐well plates and measured the absorbance at 577 nm.

### 2.5 Cell culture

The LX‐2 human hepatic stellate cell and HepG2 cells were obtained from the Institute of Biochemistry and Cell Biology (Chinese Academy of Sciences, Shanghai, China). The LX‐2 cells were cultured in DMEM (Solarbio technology) with 2% foetal bovine serum (FBS; Excell Bio).^30^ We selected HepG2 as human hepatocyte cell line.^31^, ^32^ The HepG2 cells were cultured in DMEM (Solarbio Technology) with 10% foetal bovine serum (FBS; Excell Bio). All cells were cultured at 37°C in a 5% CO_2_ incubator.

### 2.6 Cell viability and apoptosis

The LX‐2 human hepatic stellate cell and HepG2 hepatocyte cell line were seeded in 96‐well plates with DMEM, which included 2% and 10% FBS, respectively, for 24 hours. Then, we exposed the cells to different concentrations of byakangelicin for 24 hours. We used MTT Cell Proliferation and Cytotoxicity Assay Kit (Solarbio Technology) to detect the cytotoxicity. For 4‐hydroxynonenal (HNE) induction, HepG2 cell apoptosis was detected by using Annexin V‐FITC Apoptosis Detection Kit (Beyotime Biotechnology).

### 2.7 Cytokines and 4‐HNE induction

In LX‐2 hepatic stellate cell, we first changed the medium with 0.1% serum for 24 hours and then treated it with TGF‐β (5 ng/mL) for 24 hours or 30 minutes (phosphorylated protein) and PDGF‐BB (50 ng/mL) for 24 hours or 20 minutes (phosphorylated protein), respectively. For 4‐HNE induction, we added 4‐HNE to HepG2 cells for 4 hours with different concentrations. All cells were exposed to byakangelicin for 24 hours.

### 2.8 Western blot analysis

We extracted total protein from liver tissues and treated LX‐2 or HepG2 cells using radioimmunoprecipitation assay buffer (Beyotime Biotechnology), phenylmethylsulphonyl fluoride (PMSF; Solarbio Technology), and protease and phosphatase inhibitor cocktail for general use (Beyotime Biotechnology). The protein concentration was measured by using a BCA protein assay kit (Beyotime Biotechnology). We loaded 20 µg of total protein onto various percentages of sodium dodecyl sulphate‐acrylamide gels. Then, we performed electrophoresis and transfer steps. The polyvinylidene fluoride membranes were incubated in 5% skimmed milk powder with Tris‐buffered saline and Tween 20 for 1 hour. The membranes were incubated with primary antibodies overnight at 4°C. The next day, we washed the membranes three times for 10 minutes each time, and we placed the membranes into the horseradish peroxidase (HRP)–conjugated secondary antibodies. We detected the protein in membranes with Affinity^®^ ECL kit (Affinity Biosciences) before membranes were incubated at room temperature for 1 hour.

### 2.9 Quantitative real‐time PCR (qRT‐PCR)

We extracted total RNA for qRT‐PCR analysis as previously described.^33^ The total RNA was extracted from LX2 cells and liver tissue with TRIzol Reagent (Thermo Fisher Scientific). After extracting the total RNA, we reversed it to cDNA. Then, we used the SYBR GreenER qPCR SuperMix Universal (Invitrogen) to perform the qPCR steps according to the manufacturer's protocols. In LX2 cells, we used glyceraldehyde phosphate dehydrogenase (GAPDH) as the internal reference, whereas β‐actin was used as the reference gene in liver tissue. Then, we enumerated the relevant sequences: α‐SMA (M): 5′‐GCTGGTGATGATGCTCCCA‐3′ and 5‐GCCCATTCCAACCATTACTCC‐3′; Col1a1 (M): 5′‐CCAAGAAGACATCCCTGAAGTCA‐3′ and 5‐TGCACGTCATCGCACACA‐3′; β‐actin: 5′‐AGGCCAACCGTGAAAAGATG‐3′ and 5′‐AGAGCATAGCCCTCGTAGATGG‐3′; α‐SMA (H): CTATGAGGGCTATGCCTTGCC and GCTCAGCAGTAGTAACGAAGGA; Col1a1 (H): 5′‐AGGGCAACAGCAGGTTCACTTACA‐3′ and 5‐AGCGGGGGAAGGAGTTAATGAAAC‐3′; GAPDH: 5′‐GGCCCCTCTGGAAAGCTGTG‐3′ and 5′‐CCGCCTGCTTCACCACCTTCT‐3′.

### 2.10 Immunofluorescence staining

After the 24‐well plates were seeded with LX2 (or HepG2) cells for 24 hours, we changed the medium from DMEM with 2% FBS (or 10% FBS) to 0.1% FBS for 24 hours. Then, the experiment was performed according to the induction and dosing methods mentioned before. Afterwards, we used 4% paraformaldehyde for 20 minutes at room temperature to fix cells. We washed cells with PBS before they were permeabilized with 0.2% Triton X‐100 (Fluorochem) for 30 minutes and blocked with 5% bovine serum albumin in PBS for 1 hour. We also added primary antibodies to humidified chamber overnight at 4°C. The next day, we converted the primary antibodies into TRITC‐conjugated or FITC‐conjugated secondary antibodies after washing with PBS. After incubation for 2 hours, cells were counterstained using antifade mounting medium with DAPI (Beyotime Biotechnology). Similar steps were conducted for the liver tissue. We also used the One Step TUNEL apoptosis assay kit (Beyotime Biotechnology). Then, we used laser scanning confocal microscope (Leica, TCS SP8) to observe protein expression and take photos.

### 2.11 Flow cytometry

We used the Annexin V‐FITC apoptosis detection kit (Beyotime Biotechnology) to perform the test according to the instructions and test by flow cytometry (Becton, Dickinson and Company, LSR Fortessa).

### 2.12 Statistical analyses

Data were processed using GraphPad Prism software and were expressed as means ± SEM. Differences in the measured variables between experimental and control group were assessed by using Student's *t* tests. Multiple group comparisons were performed using a one‐way ANOVA with Bonferroni's multiple comparison test. *P* values of <.05 were considered to be statistically significant.

## 3 RESULTS

### 3.1 Byakangelicin treatment alleviates liver fibrosis and liver damage in carbon tetrachloride‐induced liver fibrosis mouse models

**Figure 1:**
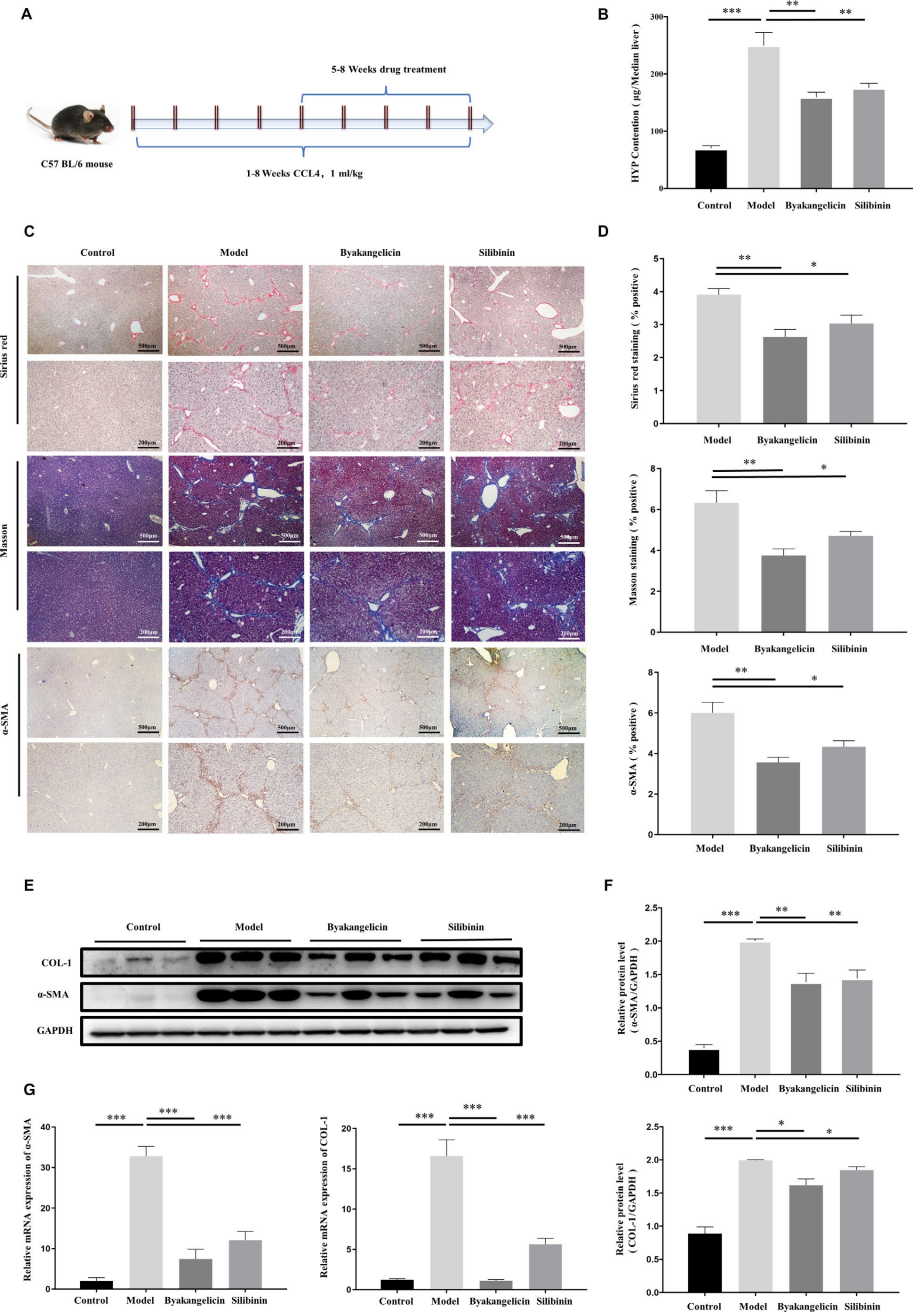
Byakangelicin treatment reduces carbon tetrachloride‐induced liver fibrosis. A, Animal liver fibrosis modelling plan. B, Detection of hydroxyproline in liver tissue. C, Classical tissue staining in liver fibrosis: Sirius red, Masson and α‐SMA staining; D, three stained quantitative areas. E, Protein content of α‐SMA and COL‐1 in liver tissue; F, corresponding grey analysis. G, Real‐time PCR analyses of α‐SMA and COL‐1 in liver tissues. For the statistics of each panel in this figure, *P < .05 vs model, **P < .01 vs model, ***P < .001 vs model, n = 3

**Figure 2:**
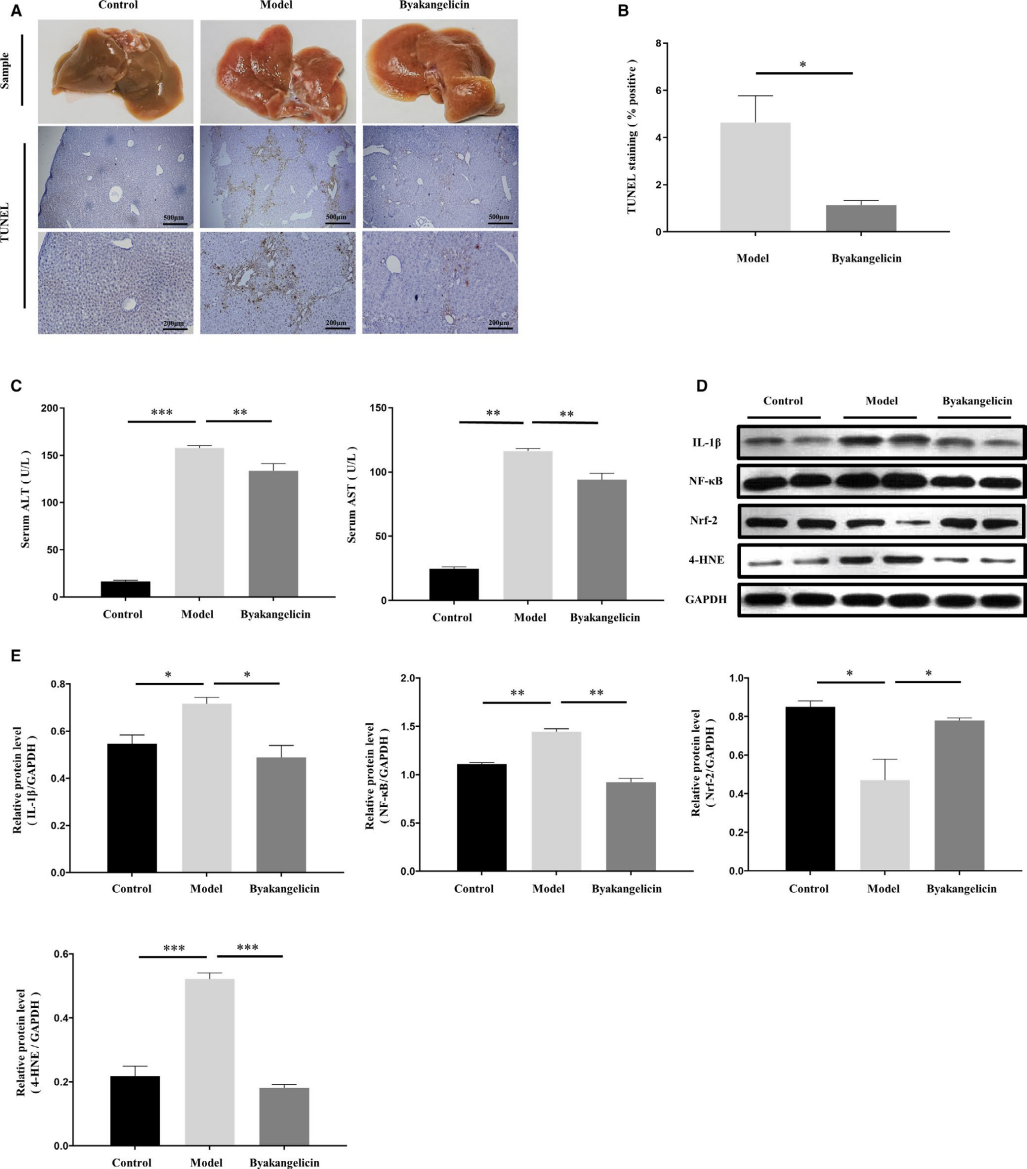
Byakangelicin alleviates liver injury in a carbon tetrachloride‐induced model. A, Liver tissue samples and evaluation of liver cell apoptosis using TUNEL staining. B, Quantitative analysis of stained area using software. C, Detection and analysis of alanine aminotransferase and aspartate aminotransferase in serum. D and E, Western blot analyses of IL‐1β, NF‐κB, Nrf‐2 and 4‐HNE in animal tissue with densitometry. For the statistics of each panel in this figure, *P < .05 vs model, **P < .01 vs model, ***P < .001 vs model, n = 3

We first evaluated the effects of byakangelicin treatment on carbon tetrachloride–induced liver fibrosis, and the experimental programme is shown in Figure 1A. In addition, we examined the levels of hydroxyproline (Figure 2B). The results showed that byakangelicin significantly reduced the accumulation of hydroxyproline. To further observe the deposition of collagen, we used dyeing methods, Masson and Sirius red staining. At the same time, the fibrosis marker α‐SMA was also reduced to some extent, whereas the anti‐fibrosis effect of byakangelicin treatment was better than that of Silibinin. Then, we further counted the fibrosis area and α‐SMA area of different staining methods (Figure 1C,D). In our statistical analysis, we determined that byakangelicin reduced carbon tetrachloride–induced liver collagen and α‐SMA deposition. Next, we tested the protein levels of α‐SMA and COL1 in liver tissue and the results indicated that byakangelicin significantly reduced the protein levels of α‐SMA and COL1 (Figure 1E,F). The qRT‐PCR results indicated the same conclusion (Figure 1G). Then, we evaluated the liver surface (Figure 2A) and hepatocyte apoptosis by TUNEL staining with quantification (Figure 2B) and analysed the serum levels of ALT and AST (Figure 2C) in different groups to evaluate liver damage. We found that compared with the model group, byakangelicin significantly reduced the degree of liver damage. We also evaluated the anti‐inflammatory and antioxidant effects of byakangelicin in CCl4‐induced hepatic injury in vivo. We extracted the protein from liver tissues and detected the expression of IL‐1β, NF‐κB, Nrf‐2 and 4‐HNE using the method of Western blot and performed greyscale analysis of Western blots (Figure 2D,E). The results showed that byakangelicin could prevent liver inflammation by down‐regulating NF‐κB expression and restore the antioxidant defence mechanism in the hepatic tissue by up‐regulating Nrf2 expression and prevent the elevation of oxidative stress product 4‐HNE. In summary, byakangelicin could significantly reduce liver fibrosis and liver damage in CCl4‐induced liver fibrosis mouse model.

### 3.2 Byakangelicin alleviates hepatic stellate cell activation by inhibiting the TGF/Smad3 signalling pathway

**Figure 3:**
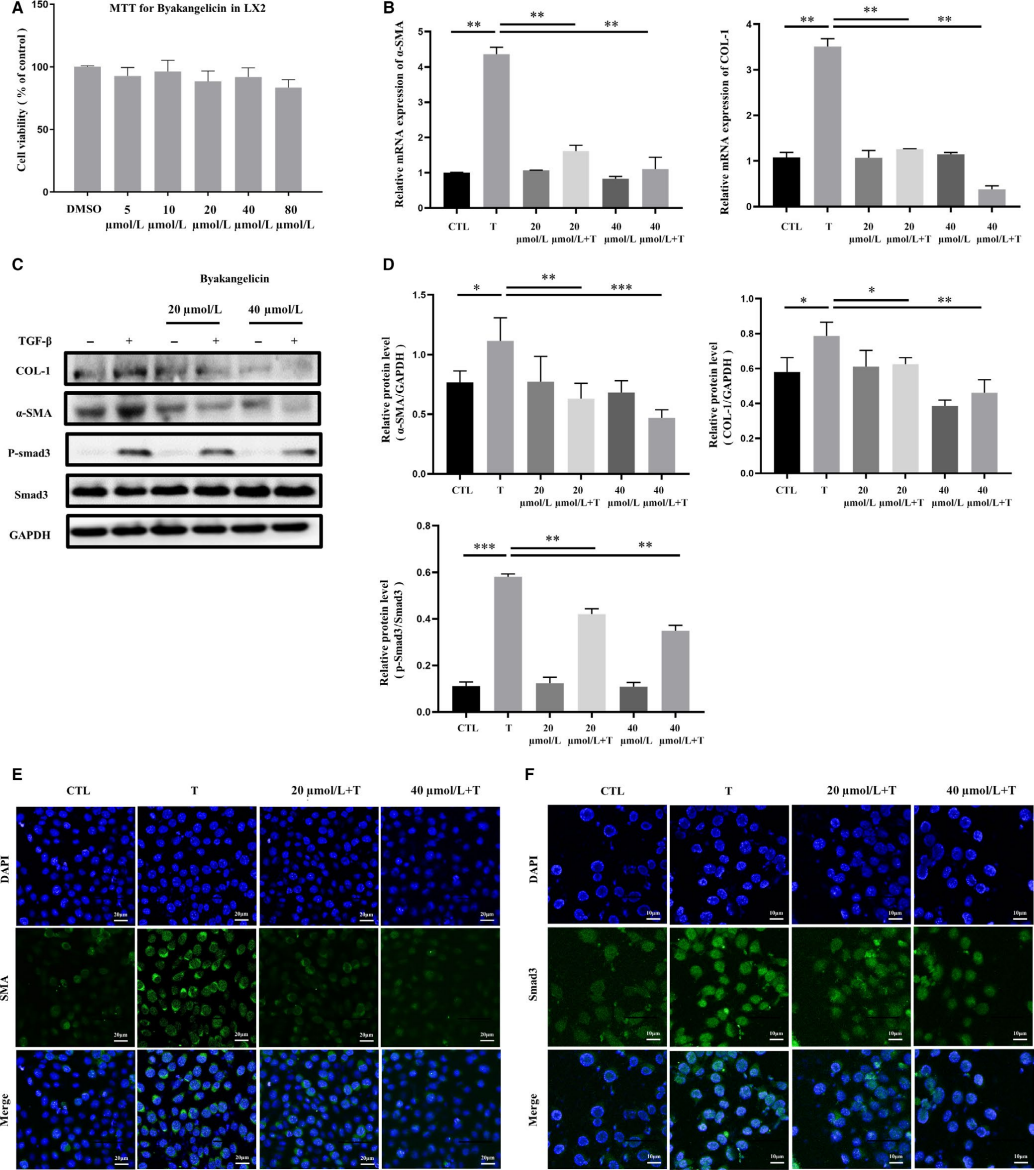
Byakangelicin inhibits the TGF/Smad3 pathway in TGF‐β‐induced activation of hepatic stellate cell. A, Detection of byakangelicin's cytotoxicity in liver stellate cell line LX2 using MTT. B, Real‐time PCR analyses of α‐SMA and COL‐1 in LX2 cells. C and D, Western blot analyses of α‐SMA, COL‐1, P‐Smad3, Smad3 and GAPDH protein expression in LX2 cells with densitometry. E and F, Immunofluorescence using antibody against α‐SMA and Smad3. For the statistics of each panel in this figure, *P < .05, **P < .01, ***P < .001, n = 3

We next studied the anti‐fibrotic activity of byakangelicin in vitro. We first examined the cytotoxicity of byakangelicin on hepatic stellate cell LX‐2 (Figure 3A). According to the pre‐experiment results, two concentrations (20 and 40 µmol/L) were selected. We examined the mRNA levels of α‐SMA and COL‐1 in TGF‐activated‐hepatic stellate cell and found that byakangelicin could inhibit hepatic stellate cell activation in a dose‐dependent manner (Figure 3B). In addition, byakangelicin also significantly reduced the expression of fibrotic marker proteins (α‐SMA and COL‐1) at the protein level. Interestingly, we also found that the downstream Smad3 signalling pathway of TGF‐β is also inhibited by byakangelicin (Figure 3C). We also performed a greyscale analysis of protein expression (Figure 3D). In order to more intuitively observe the anti‐fibrotic effect of byakangelicin, we used immunofluorescence to observe the cells in the visual field, and the same results were obtained (Figure 3E,F). In conclusion, these results show that byakangelicin alleviates TGF‐induced hepatic stellate cell activation by inhibiting Smad3 phosphorylation.

### 3.3 Byakangelicin inhibits PDGF‐induced hepatic stellate cell proliferation and activation

**Figure 4:**
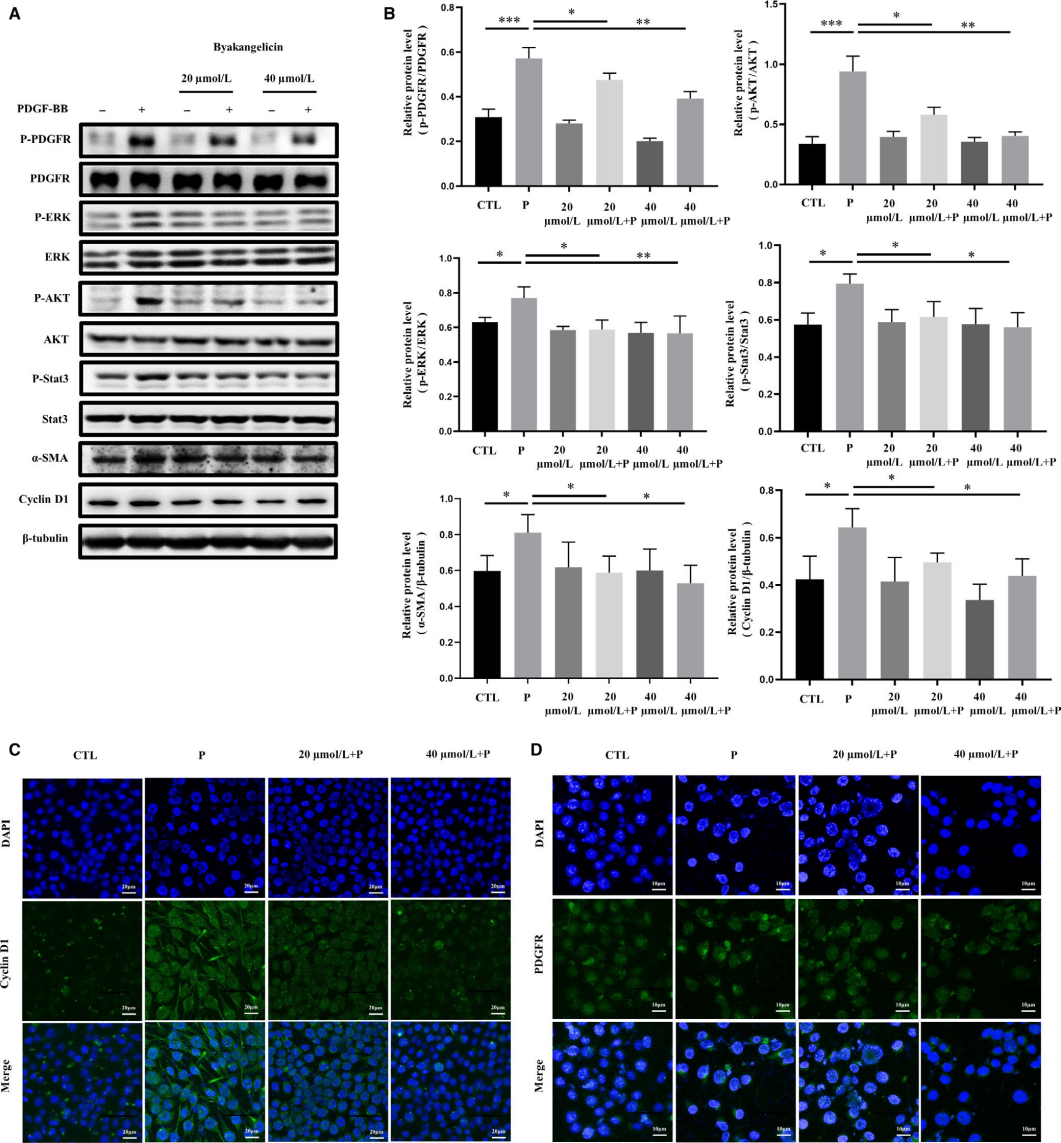
Byakangelicin inhibits the proliferation and activation of PDGF‐induced hepatic stellate cell by inhibiting PDGFR/ERK, PDGFR/AKT, and PDGFR/Stat3. A and B, Western blot analyses of P‐PDGFR, PDGFR, P‐ERK, ERK, P‐AKT, AKT, P‐Stat3, Stat3, α‐SMA and cyclin D1 protein expression with densitometry. C and D, Immunofluorescence by using antibody against cyclin D1 and PDGFR. For the statistics of each panel in this figure, *P < .05, **P < .01, ***P < .001, n = 3

PDGF, as the most important mitogen of hepatic stellate cell, is very important in the progress of liver fibrosis.^34^ To this end, we used PDGF to activate hepatic stellate cell and test the effect of byakangelicin. Firstly, we used Western blot analysis to detect two activation markers, α‐SMA and Cyclin D1, and detect the effect of byakangelicin on the proliferation and activation of hepatic stellate cell. The results demonstrated that byakangelicin affected PDGF‐induced expression of α‐SMA and cyclin D1. We further determined that byakangelicin can inhibit the phosphorylation of PDGFR to a certain extent in a dose‐dependent manner. In addition, ERK/Akt/Stat3, a downstream pathway of PDGF related to liver fibrosis, was inhibited by byakangelicin (Figure 4A). On this basis, we also performed quantitative greyscale analysis, and the results were consistent with the observations of Western blot analysis (Figure 4B). We used immunofluorescence to observe significant changes in the administration group compared with the PDGF‐induced group to clearly observe the effect of byakangelicin on cyclin D1 and PDGFR. Results show that byakangelicin does inhibit the expression of cyclin D1 protein and the activation of PDGFR (Figure 4C,D). In summary, byakangelicin could inhibit PDGF‐induced hepatic stellate cell proliferation and activation.

### 3.4 Byakangelicin attenuates 4‐HNE–induced hepatocyte apoptosis

**Figure 5:**
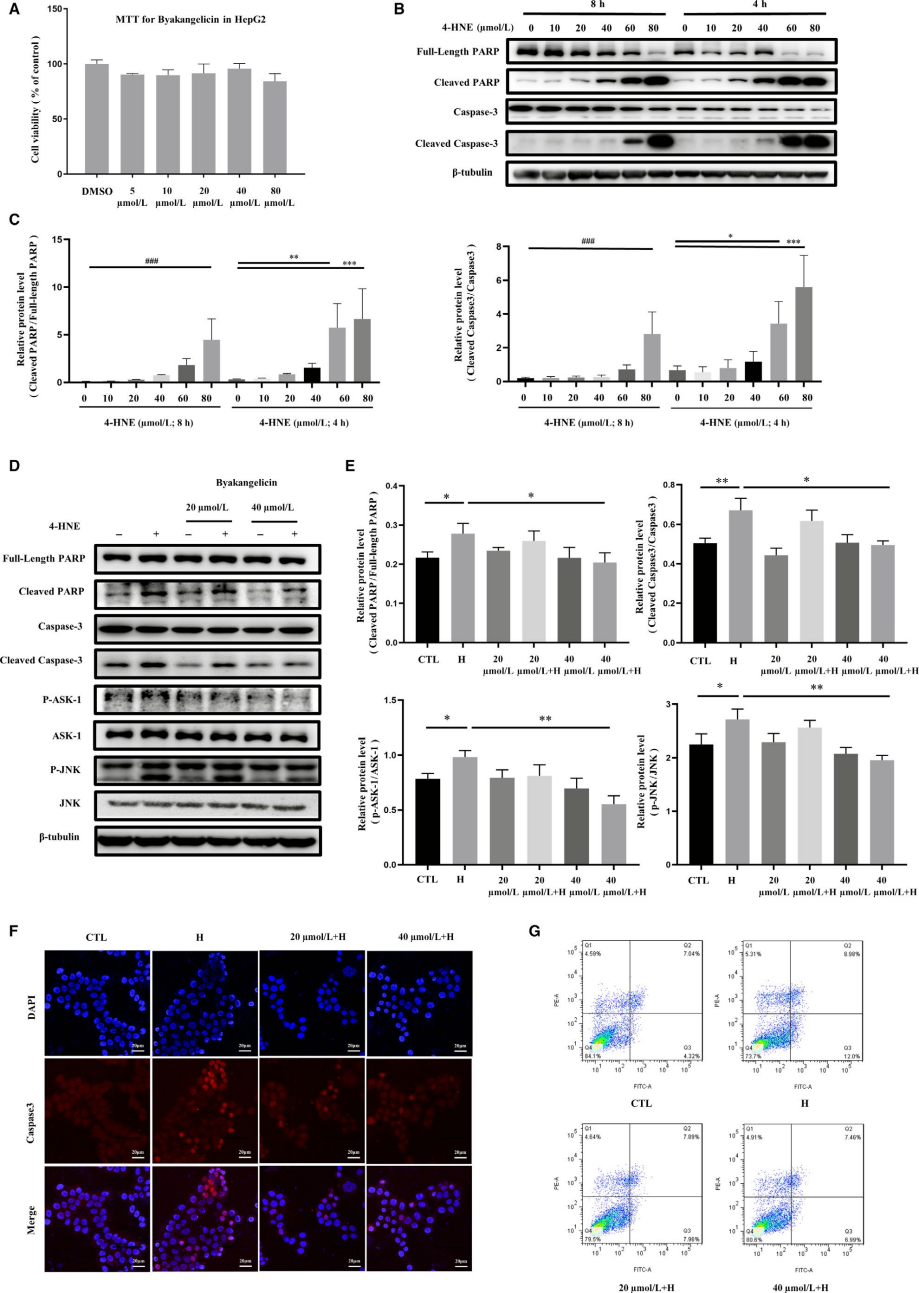
Byakangelicin inhibits 4‐HNE–induced hepatocyte apoptosis by inhibiting ASK1/JNK pathway. A, Detection cytotoxicity of byakangelicin in liver stellate cell line LX2 using MTT. B and C, Different time and concentration gradients to detect 4‐HNE–induced hepatocyte HepG2 apoptosis protein full‐length PARP, PARP, cleaved caspase‐3, caspase‐3 and internal reference protein β‐tubulin expression with densitometry. D and E, Protein full‐length PARP, PARP, cleaved caspase‐3, caspase‐3, JNK, P‐JNK, ASK‐1 and P‐ASK‐1 were also detected and quantified using Western blot analyses. F, Immunofluorescence by using antibody against caspase‐3. G, Apoptosis detection by using flow cytometry. For the statistics of each panel in this figure, ^#^ *P* < .05, ^##^ *P* < .01, ^###^ *P* < .001; *P < .05, **P < .01, ***P < .001, n = 3

Based on the above animal experiment results, we found that byakangelicin significantly reduced the expression of 4‐HNE associated with liver injury, and we further conducted a more in‐depth study of the mechanism of byakangelicin. We first tested the cytotoxicity of byakangelicin on hepatocyte HepG2 cells and found no obvious toxicity (Figure 5A). Based on previous studies,^35^ we analysed the expression of full‐length PARP, cleaved PARP, caspase‐3 and cleaved caspase‐3, by Western blot after 4 and 8 hours exposure to different concentrations (0‐80 μmol/L) of 4‐HNE. We performed time‐course stimuli experimental designs to find the appropriate stimulus time and concentration of 4‐HNE (Figure 5B,C). We stimulated apoptosis according to the selected time and concentration of 4‐HNE and exposed HepG2 to byakangelicin. We found that byakangelicin (40 µmol/L) could significantly attenuate the expression of cleaved PARP and cleaved caspase‐3, and we also observed that byakangelicin could inhibit 4‐HNE–induced ASK‐1/JNK signalling pathway (Figure 5D,E). We further used immunofluorescence method for caspase‐3 (Figure 5F) and flow cytometry (Figure 5G) to obtain the results consistent with Western blot analysis. High concentration of byakangelicin significantly inhibited ASK‐1/JNK signalling and weakened the apoptosis‐promoting effect of 4‐HNE.

## 4 DISCUSSION

In this study, the inhibitory effect of byakangelicin on the progression of liver fibrosis was investigated. First, the inhibitory effects of byakangelicin on liver fibrosis and liver injury were verified from animal models. Then, we conducted cell experiments to explore the mechanism of drug action. We studied the activation of hepatic stellate cell by exploring the signalling pathways of two major pro‐fibrotic factors, TGF and PDGF. We used a 4‐HNE–induced hepatocyte apoptosis model to study the mechanism of byakangelicin on protection of hepatocytes.

Carbon tetrachloride induction is one of the most commonly used in animal models of liver fibrosis studies.^36^, ^37^, ^38^ Liver tissue and blood samples were obtained after the experiment. COL‐1 and α‐SMA were tested in various ways to evaluate the degree of fibrosis, which is an important marker protein of liver fibrosis. We also carried out tissue sections to determine the collagen content. We found that byakangelicin has a better effect of alleviating liver fibrosis comparing the positive drug Silibinin. We choosing silibinin as a positive drug according to related articles, the author used silymarin (the main ingredient is silibinin) to perform an intragastric test, proving that it has anti‐liver fibrosis ability, but there is indeed a problem of limited bioavailability.^39^, ^40^ Although some studies have conducted intravenous injections in human trials, we can see that most animal experiments still use intragastric administration,^27^, ^41^, ^42^, ^43^, ^44^, ^45^ and only to explore the improvement of drug properties, intravenous administration was used in trials,^46^ and some trials used the method of silibinin‐phosphatidylcholine‐vitamin E complex,^47^ but these studies are few and not widely used. Therefore, we believe that the more stable method of using silibinin in animal experiments is still intragastric administration. Subsequent further exploration experiments can be studied by intravenous administration. We further examined the levels of ALT and AST in the blood and performed TUNEL staining to determine liver damage. Also, we detected inflammation‐related proteins and found that byakangelicin can alleviate the negative effects of carbon tetrachloride. It is worth noting that the content of 4‐HNE decreased significantly compared with the model group. Anyway, the animal results showed that byakangelicin could alleviate liver damage and reduce the progress of liver fibrosis.

Hepatic stellate cells have been widely studied because their activation is a key event in liver fibrosis.^48^, ^49^, ^50^ Therefore, inhibition of cell activation in hepatic stellate cell is one of the most important treatments. There are many signal pathways closely related to the activation of hepatic stellate cell, but the most important ones are the TGF‐ and PDGF‐related signal pathways. TGF‐β is a key pro‐fibrotic factor. In many diseases, TGF‐β is one of the most critical factors for fibrosis‐related diseases. Therefore, in addition to the necessary animal efficacy tests, we also focused on the detection of cell signalling pathways. We first tested the effect of white angelica on this pathway. The results showed that for the classic Smad3 pathway, byakangelicin indeed plays a relevant role, reducing the phosphorylation signal transmission and TGF‐β–induced stellate cell activation by detecting related phenotypic proteins. Similarly, PDGF is also an important pro‐fibrotic factor, with strong pro‐proliferative activity, which is essential for the proliferation and activation of hepatic stellate cell. We determined that byakangelicin can inhibit the phosphorylation of PDGF downstream signalling pathway proteins PDGFR, AKT, ERK, and STAT3 and cyclin D1. byakangelicin attenuates the classic TGF‐β/Smad3 signal transmission and the phosphorylation of PDGFR. Therefore, inhibiting the downstream signal transduction of PDGF is important to prevent the activation of hepatic stellate cell.

In addition, many studies have shown that liver cell apoptosis is highly correlated with liver fibrosis and that lipid peroxidation is an important factor in the occurrence of liver fibrosis and subsequent damage and apoptosis of liver cells; 4‐HNE is an important lipid peroxidation product and has the effect of inducing apoptosis.^51^, ^52^ Many investigations reported that 4‐HNE has an important influence on the development of liver fibrosis.^53^ Recent studies have shown that 4‐HNE can induce apoptotic signal transduction through a variety of pathways related to ASK‐1 and JNK activation.^54^ We observed in this experiment that byakangelicin has a certain inhibitory effect on 4‐HNE–induced hepatocyte apoptosis. Previous studies have confirmed that byakangelicin has a certain anti‐inflammatory activity. Our studies confirmed that byakangelicin can simultaneously inhibit the progression of liver fibrosis and can reduce liver cell apoptosis to a certain extent, which reduces liver damage. Moreover, it has a good inhibitory effect on the important signalling pathways of hepatic stellate cell activation in response to key events in liver fibrosis. byakangelicin also plays an important role and attenuates ASK‐1 and JNK signalling in the death receptor pathway. In our study, HepG2 was used as a cell line of liver cells for apoptosis test, which was indeed not comprehensive enough. Additional instructions on liver cell lines could be made in the future.

**Figure 6:**
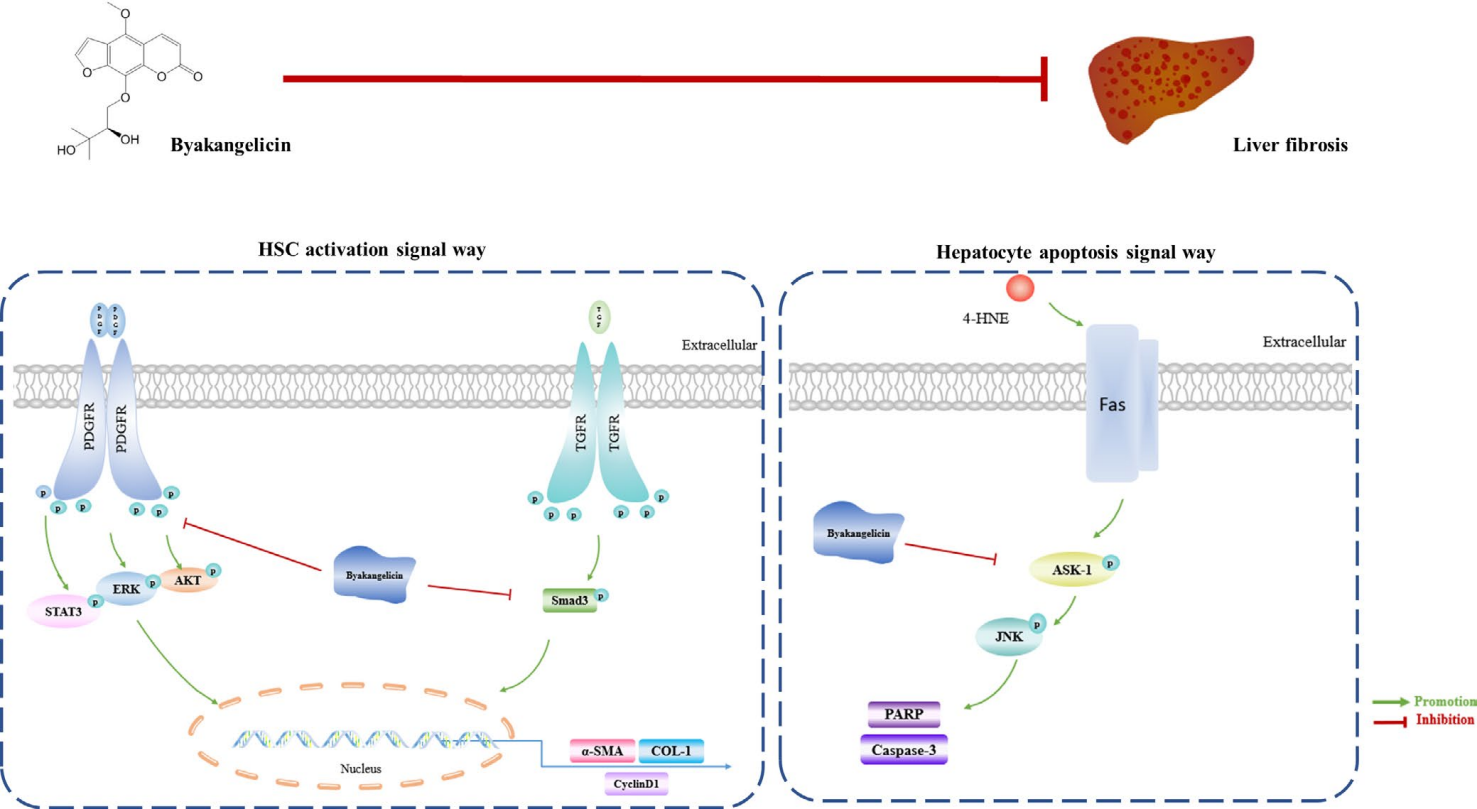
Mechanism diagram of byakangelicin attenuating the progression of liver fibrosis. byakangelicin inhibits the proliferation and activation of hepatic stellate cell by inhibiting the TGF/Smad3 signalling pathway and PDGF‐ERK, AKT and Stat3 signalling. In addition, byakangelicin could inhibit 4‐HNE–induced apoptosis and reduce death signalling through the ASK/JNK pathway in hepatocytes

In conclusion, our study indicated that byakangelicin could inhibit the progression of liver fibrosis by inhibiting the activation of hepatic stellate cell via regulating the TGF‐β and PDGF signalling pathway and protecting hepatocytes from apoptosis via regulating the ASK/JNK pathway (Figure 6), which provides ideas for the development of potential drugs and treatment methods of liver fibrosis.

## AUTHOR CONTRIBUTION

**Xiaohe Li:** Conceptualization (lead); Data curation (equal); Formal analysis (equal); Investigation (equal); Methodology (lead); Project administration (lead); Resources (equal); Software (equal); Supervision (lead); Validation (equal); Visualization (lead); Writing‐original draft (equal); Writing‐review & editing (equal). **Shuaibo Shao:** Conceptualization (lead); Data curation (equal); Formal analysis (equal); Investigation (lead); Methodology (lead); Project administration (equal); Resources (lead); Software (equal); Supervision (equal); Validation (equal); Visualization (equal); Writing‐original draft (equal); Writing‐review & editing (equal). **Hailong Li:** Conceptualization (supporting); Data curation (supporting); Formal analysis (supporting); Investigation (equal); Methodology (equal); Project administration (equal); Resources (supporting); Software (equal); Supervision (equal); Validation (equal); Visualization (equal); Writing‐original draft (supporting); Writing‐review & editing (supporting). **Zhun Bi:** Conceptualization (equal); Investigation (supporting); Methodology (equal); Project administration (supporting); Resources (supporting); Software (supporting); Supervision (equal); Validation (equal); Visualization (equal). **Shanshan Zhang:** Conceptualization (supporting); Investigation (equal); Methodology (equal); Resources (supporting); Validation (equal); Visualization (equal). **Yiying Wei:** Conceptualization (equal); Investigation (equal); Methodology (supporting); Resources (supporting); Validation (equal). **Jiakun Bai:** Conceptualization (supporting); Methodology (supporting); Software (supporting); Validation (supporting). **Ruotong Zhang:** Formal analysis (supporting); Investigation (supporting); Methodology (supporting). **Xiaoyang Ma:** Investigation (supporting); Methodology (supporting); Resources (supporting); Software (supporting). **Bowei Ma:** Investigation (supporting); Resources (supporting); Validation (supporting). **Liang Zhang:** Conceptualization (supporting); Formal analysis (supporting); Methodology (supporting). **Chunfeng Xie:** Conceptualization (supporting); Investigation (supporting); Methodology (supporting); Resources (supporting). **Wen Ning:** Conceptualization (supporting); Investigation (supporting); Methodology (supporting); Project administration (supporting); Resources (equal); Software (supporting). **Honggang Zhou:** Conceptualization (equal); Funding acquisition (equal); Investigation (equal); Methodology (equal); Project administration (equal); Resources (equal); Software (equal); Supervision (equal); Validation (equal); Visualization (equal). **Cheng Yang:** Conceptualization (equal); Funding acquisition (lead); Investigation (equal); Methodology (equal); Project administration (equal); Resources (equal); Software (supporting); Supervision (supporting); Validation (supporting); Visualization (supporting).

## Data Availability

Data are available on request from the authors.
